# Efficacy and safety of RTS, S and R21 malaria vaccines in children under five in Africa: a systematic review and meta-analysis of randomized controlled trials

**DOI:** 10.1186/s12936-026-05954-5

**Published:** 2026-05-27

**Authors:** Khulud Mahmood Nurani, Najib Kadernani, Emmanuel Korir, Merna Akram Estreed, Arkadeep Dhali, Rugut Clinton, Khalid Kadernani, Vincent Kipkorir, Pius Omullo, Moses Masika

**Affiliations:** 1 Faculty of Health Sciences, University of Nairobi, Nairobi, Kenya; 2 School of Medicine, Dentistry and Biomedical Sciences, Queen’s University, Belfast, UK; 3 Department of Medicine, The Nairobi West Hospital, Nairobi, Kenya; 4 College of Surgeons of East, Central and Southern Africa, Arusha, Tanzania; 5 Department of Clinical Services, St. Teresa’s Hospital, Kiambu, Kenya; 6 Department of Medical Microbiology, University of Nairobi, Nairobi, Kenya

## Abstract

**Background:**

Malaria remains a major public health burden in Africa, particularly among children under five years of age. By consolidating findings across randomized controlled trials on the RTS,S and R21 malaria vaccines, this study seeks to provide an evidence based guide.

**Methods:**

We conducted a systematic review and meta-analysis of studies of randomized controlled trials evaluating the RTS,S and R21 malaria vaccines in African children under five. Data sources included PubMed, Scopus, and Embase. Primary outcomes were the vaccine efficacies against first/only episodes of malaria, multiple episodes and severe malaria; and the incidence of severe adverse events following vaccination. Pooled effect estimates were calculated using random-effects models.

**Results:**

Eleven studies enrolling 27,178 participants (RTS,S: 21,589; R21: 5589) were included. For RTS,S, pooled efficacy against first clinical malaria episode was 32% (HR 0.68, 95% CI 0.61–0.76; I^2^ = 0%), against multiple episodes was 29% (IRR 0.71, 95% CI 0.55–0.91; I^2^ = 11.3%), and against severe malaria was 17–22% (RR 0.78–0.83). The R21 vaccine demonstrated 68% efficacy against first episodes (HR 0.32, 95% CI 0.26–0.39) and 67% against multiple episodes (IRR 0.33, 95% CI 0.27–0.41) in a single phase 3 trial. Pooled SAE incidence was 11.2% (95% CI 6.6–18.6%; I^2^ = 96.2%) for RTS,S and 3% (95% CI 1–6%; I^2^ = 80.6%) for R21, with no vaccine-attributable deaths. Both vaccines showed acceptable reactogenicity profiles.

**Conclusion:**

Both RTS,S and R21 vaccines demonstrate protective efficacy against clinical malaria in African children under five, with R21 showing higher efficacy in initial trials. RTS,S provides moderate, reproducible protection in clinical trials. Both vaccines have acceptable safety profiles, though continued pharmacovigilance is essential.

## Introduction

Malaria remains one of the leading causes of morbidity and mortality in sub-Saharan Africa, disproportionately affecting children under five years of age. According to the World Health Organization (WHO), there were an estimated 282 million malaria cases and 610,000 deaths globally in 2024, with approximately 94% of all cases and 95% of all deaths occurring in Africa [1]. Children below the age of five accounted for just over 75% of all malaria deaths, signifying their vulnerability to this disease [1].

For decades, malaria prevention has relied heavily on vector control measures such as insecticide-treated nets (ITN) and indoor residual spraying (IRS), as well as chemoprevention strategies [2, 3]. While these approaches have contributed to substantial reductions in malaria burden, their effectiveness has been undermined by insecticide resistance, inconsistent use of ITNs, suboptimal coverage of IRS, and persistent transmission in high-endemicity regions [3]. Consequently, the development of safe and effective vaccines has long been considered a priority in the fight against malaria.

The RTS,S/AS01 (Mosquirix) vaccine, developed by GlaxoSmithKline, became the first malaria vaccine to receive a positive recommendation from the WHO in 2021 for broader use among African children [4]. RTS,S and R21 are both pre-erythrocytic malaria vaccines targeting the circumsporozoite protein (CSP) of Plasmodium falciparum, but they differ in formulation and immunogenicity. RTS,S is a recombinant virus-like particle composed of the central repeat and C-terminal regions of CSP fused to hepatitis B surface antigen (HBsAg), co-expressed with unfused HBsAg and formulated with the AS01 adjuvant system to enhance immune activation [5]. In contrast, R21 is a more compact virus-like particle that contains a higher proportion of CSP fused to HBsAg, without excess unfused HBsAg, and is adjuvanted with Matrix-M, a saponin-based nanoparticle that promotes strong antibody and T-cell responses [6]. These structural and adjuvant differences result in variations in anti-CSP antibody titers and durability of immune protection, which may partly explain observed differences in vaccine efficacy across clinical trials.

Large-scale trials demonstrated moderate efficacy against clinical malaria, although protection waned over time, necessitating booster doses. More recently, the R21/Matrix-M vaccine, developed by the University of Oxford in collaboration with the Serum Institute of India, has shown promising efficacy results in early and late-phase clinical trials, with some evidence of higher and more sustained protection compared to RTS,S [7].

Despite these advances, uncertainties remain regarding the comparative efficacy and safety of RTS,S and R21, especially across diverse malaria-endemic regions of Africa. This systematic review and meta-analysis thus aims to synthesize available evidence on the efficacy and safety profiles of RTS,S and R21 malaria vaccines in children under five years of age in Africa. By consolidating findings across randomized controlled trials, we seek to provide a robust evidence base to guide policymakers, clinicians, and global health advocates in malaria prevention strategies.

## Methods

This systematic review and meta-analysis was conducted following the Preferred Reporting Items for Systematic Reviews and Meta-Analyses (PRISMA 2020) guidelines [8]. The review protocol was prospectively registered in the International Prospective Register of Systematic Reviews, PROSPERO Registration number CRD42024619568.

### Data sources and search strategy

A systematic literature search was performed by two authors (AD and KN) using PubMed/MEDLINE, Scopus and Embase databases to identify eligible studies. All records up to 11th February, 2025, the latest search date, were included. Search terms used for all databases are shown below (Table 1). No restrictions on date, language, and study type were specified when searching the databases. MeSH terms were utilized to incorporate key indexing functions within PubMed. Backward and forward citation tracking was used in order to include relevant articles as appropriate. Duplicate citations were removed using Rayyan and cross-checked manually [9]. Articles that met the inclusion criteria had their title and abstract screened for relevance. Full text records were extracted for formal quantitative and qualitative analysis.

**Table 1:** Search terms

Database	Search terms
Pubmed	( ("Child"[Mesh] OR "Infant"[Mesh] OR child*[tiab] OR infant*[tiab] OR "under five"[tiab])) AND ( "Africa South of the Sahara"[Mesh] OR "Africa"[Mesh] OR "sub-Saharan Africa"[tiab]) AND ( "Malaria Vaccines"[Mesh] OR "RTS,S"[tiab] OR R21[tiab] OR "Plasmodium falciparum vaccine"[tiab]) AND ( "Vaccine Efficacy"[Mesh] OR efficacy[tiab] OR effectiveness[tiab] OR "Treatment Outcome"[Mesh]) AND ( "Drug-Related Side Effects and Adverse Reactions"[Mesh] OR "Safety"[Mesh] OR "adverse events"[tiab] OR "safety profile"[tiab])
Scopus	TITLE-ABS-KEY( ( child* OR infant* OR "under five") AND ( africa OR "sub-Saharan Africa") AND ( "malaria vaccine*" OR "RTS,S" OR r21 OR "Plasmodium falciparum vaccine") AND ( efficacy OR effectiveness OR "treatment outcome") AND ( "drug-related side effects" OR safety OR "adverse events" OR "safety profile"))
Embase	('child'/de or 'infant'/de or child*.mp. or children.mp. or infant*.mp. or "under five".mp.) and ('africa'/de or 'africa, sub-saharan'/de or "sub-saharan africa".mp.) and ('malaria vaccine'/de or "RTS,S".mp. or R21.mp. or "plasmodium falciparum vaccine".mp.) and ('vaccine efficacy'/de or efficacy.mp. or effectiveness.mp. or 'treatment outcome'/de) and ('drug related side effect'/de or 'safety'/de or "adverse events".mp. or "safety profile".mp.) [mp = title, abstract, heading word, drug trade name, original title, device manufacturer, drug manufacturer, device trade name, keyword heading word, floating subheading word, candidate term word]

### Eligibility criteria and article screening

We included randomized controlled trials evaluating RTS,S or R21 malaria vaccines in children under five years in Africa. Only randomized controlled trials (RCTs) were included because they provide the highest level of evidence for estimating vaccine efficacy. We excluded studies in populations older than five, studies outside Africa, non-English publications, reviews, commentaries, gray literature, and conference abstracts. Abstract screening was conducted independently by two reviewers (KK and ME), with discrepancies resolved by consensus with a third reviewer (KN). Full text screening was also conducted independently by two reviewers (KN and KK), with discrepancies resolved by consensus.

### Data extraction and study outcomes

All articles which passed screening and inclusion were considered for formal analysis. A standardized extraction form was used. Data extraction was conducted by two independent reviewers (KN and NA) and any variances resolved by consensus with a third reviewer (EK). Data extracted included study characteristics, population demographics, sample size, vaccine type, dosage, schedule, average duration of follow-up, comparator groups, and outcomes. The primary outcomes for the study were efficacy and safety of RTS, S and R21 vaccines with the PIO framework as follows (Table 2).

**Table 2:** PIO framework

PIO framework	Component
Population (P)	Children under five years of age in Africa
Intervention (I)	RTS,S or R21 malaria vaccine
Outcome (O)	Efficacy and safety

Efficacy was categorized into three outcomes: reduction in incidence of uncomplicated clinical malaria, reduction in incidence of severe malaria, and malaria-related mortality, and meta-analysis run using hazard ratios and risk ratios. Safety was measured using the frequency and severity of local and systemic adverse events.

### Data summary and synthesis

A summary of findings was generated for qualitative data. All quantitative analyses were performed in R (version 4.3) using the metafor and meta packages. For studies that only reported subgroup specific outcomes, absolute counts were added to generate combined estimates. Effect measures were then pooled using random-effects models (restricted maximum likelihood, REML) to account for between-study variability. Hazard ratios (HRs), risk ratios (RRs) and incidence risk ratios (IRRs) were transformed prior to pooling and back-transformed for presentation. Separate meta-analyses were conducted for HRs, IRRs and RRs of malaria. Statistical heterogeneity was assessed using the I^2^ statistic, τ^2^, and Cochran’s Q test, with I^2^ values of 25%, 50%, and 75% representing low, moderate, and high heterogeneity, respectively. Sensitivity analyses included leave-one-out procedures and alternative τ^2^ estimators (DerSimonian–Laird).

For RTS,S efficacy, separate meta-analyses were conducted for HRs, IRRs and RRs of malaria. Results for R21 efficacy were summarized narratively. Since no head-to-head trials or valid indirect comparisons (to facilitate network meta-analysis) were conducted, comparative conclusions were not drawn. Severe adverse events (SAEs) were pooled across all RTS,S trial arms, with results expressed as proportions and 95% confidence intervals.

### Quality and risk of bias assessment

The Cochrane Risk of Bias 2.0 tool for RCTs was used to assess the quality of included trials across domains: randomization process, deviations from intended interventions, missing outcome data, measurement of outcomes, selection of reported results. Each domain was rated as low risk, some concerns, or high risk. Quality and risk of bias were independently assessed by two authors (KN and NA).

## Results

### Search results

**Fig. 1:**
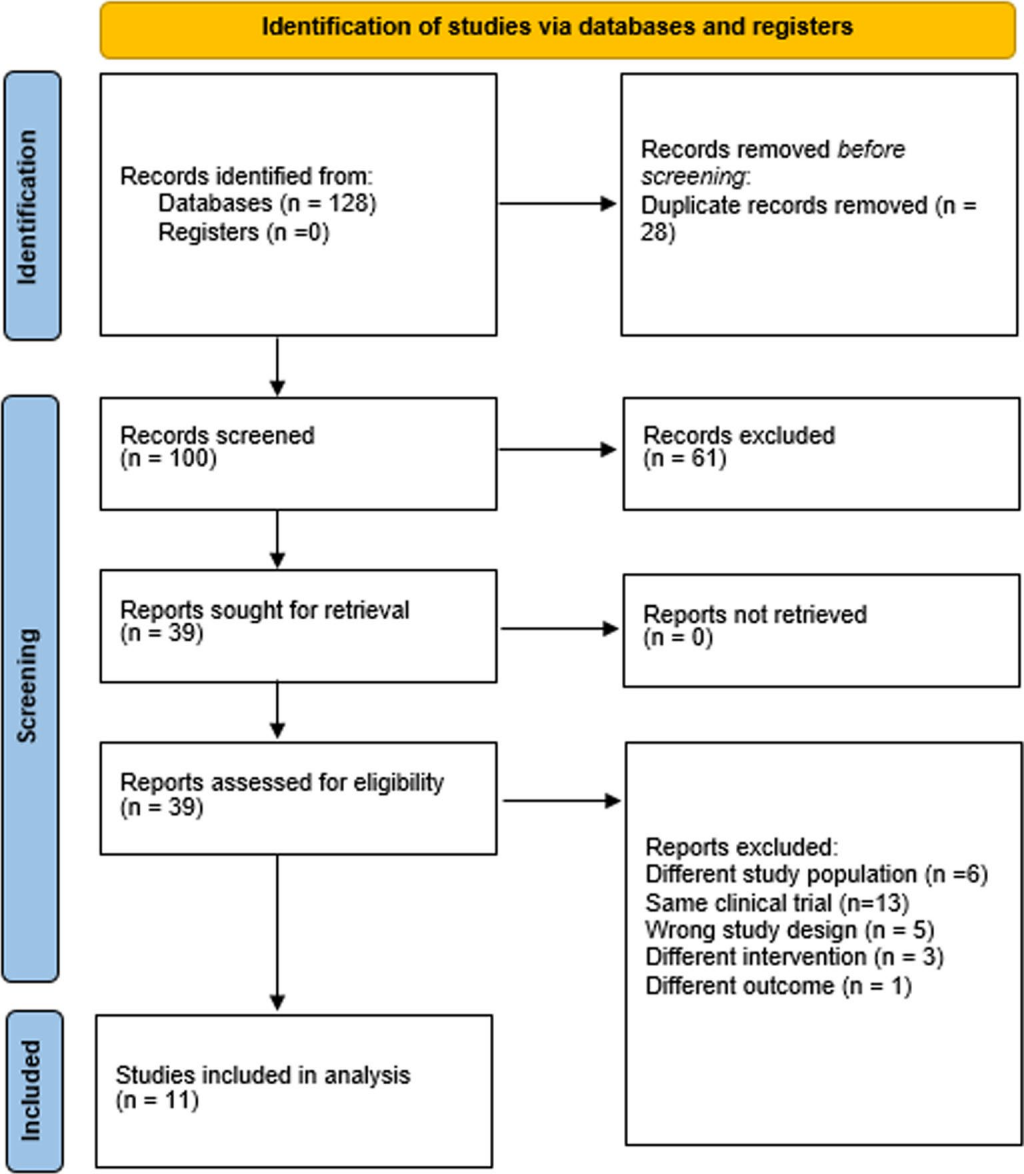
PRISMA chart illustrating study selection

We included 11 studies (published up to 2024) that enrolled 27,178 participants (RTS, S = 21,589; R21 = 5589) (Fig. 1).

### Study characteristics

Table 3 summarizes the key features of the included studies done across nine countries in sub-Saharan Africa. Eight trials evaluated the RTS,S/AS01 vaccine, administered according to three broad schedules: standard 0, 1, 2 months (with or without a 20-month booster), infant-only EPI schedules at 6–18 weeks, or fractional-dose regimens. Two trials tested the R21/Matrix-M vaccine using a 0, 1, 2-month primary series plus a 12-month booster. Follow-up ranged from 12 to 48 months, allowing assessment of both short-term efficacy and longer-term safety. All studies were conducted in high-transmission settings in sub-Saharan Africa.

**Table 3:** Summary of study characteristics

First author	Year	Country	RCT Registration No	Sample size	Age group at enrollment	Vaccine type	Vaccine schedule	Follow-up period
Abdulla [10]	2013	Tanzania	NCT00289185	340	6–10 weeks	RTS,S	8, 12 and 16 weeks of age	20 months
Aide [11]	2010	Mozambique	NCT00197028	214	6 -12 weeks	RTS,S	10, 14 and 18 weeks of age	14 months
Asante [12]	2011	Ghana, Tanzania and Gabon	NCT00436007	511	6–10 weeks	RTS,S	6 weeks, 10 weeks and 14 weeks and 6 weeks, 10 weeks and 9 months	19 months
Datoo [13]	2022	Burkina Faso	NCT03896724	450	5–17 months	R21	0, 1 and 2 months + booster after 12 months	24 months
Datoo [14]	2024	Burkina Faso, Mali, Kenya and Tanzania	NCT04704830	5139	5–36 months	R21	0, 1 and 2 months + booster after 12 months	12 months
Lusingu [15]	2010	Kenya and Tanzania	NCT00380393	894	5–17 months	RTS,S	0, 1 and 2 months	14 months
Olotu [16]	2011	Kenya and Tanzania	NCT00380393	894	5–17 months	RTS,S	0, 1 and 2 months	12 months
Owusu-Agyei [17]	2009	Ghana	NCT00360230	540	5–17 months	RTS,S	0, 1 and 2 months	19 months
RTS,S Clinical Trials Partnership [18]	2015	Kenya, Ghana, Mozambique, Tanzania, Burkina Faso, Malawi,Gabon	NCT00866619	15459	6–12 weeks and 5–17 months	RTS,S	0, 1, 2 + booster at 20 m (R3R) 0, 1, 2 (no booster, R3C) 0, 1, 2 + booster at 20 m (R3R) 0,1,2 (no booster, R3C)	48 months
Sacarlal [19]	2009	Mozambique	NCT00197041	2022	1–4 years	RTS,S	0, 1 and 2 months	45 months
Samuels [20]	2022	Ghana and Kenya	NCT03276962	1609	5–17 months	RTS,S	full doses0,1,2 and 20 months (group R012-20) full doses 0, 1, 2, 14, 26, and 38 months (R012-14), full doses 0,1 fractional doses 2,14, 26, and 38 months (Fx012-14), full doses 0,1 fractional doses at months 7, 20, and 32 (Fx017-20)	20 months

### Efficacy of the malaria vaccines

#### Efficacy of RTS,S vaccine against first or only clinical-episode (single-episode analysis)

**Fig. 2:**
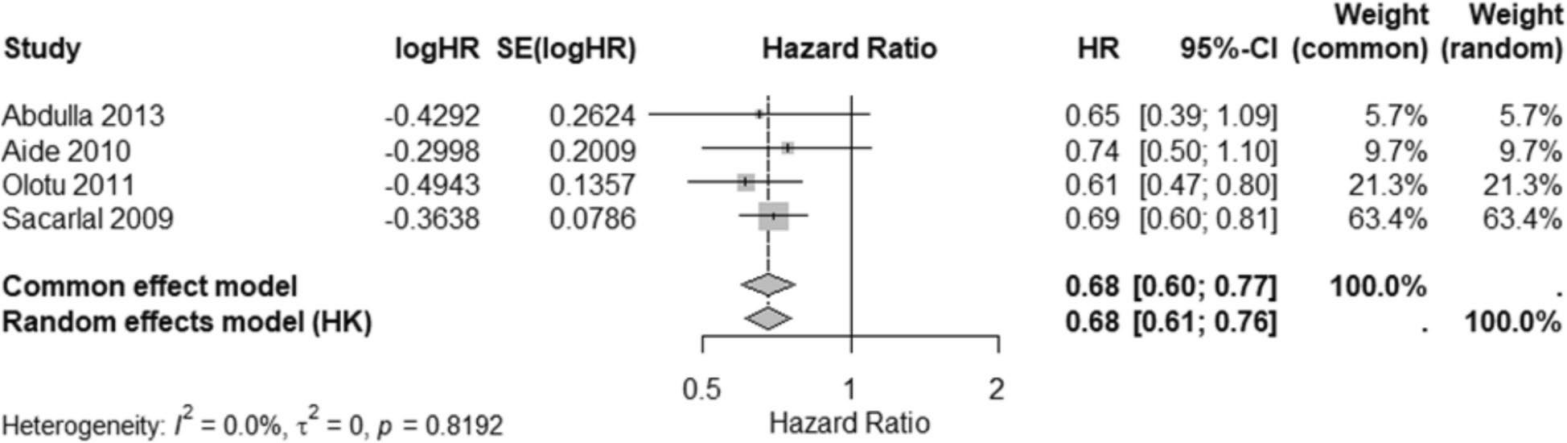
Forest plot of hazard ratios (HRs) for malaria against first clinical-episode comparing vaccinated and control groups across included randomized controlled trials. Pooled estimates were calculated using fixed-effect and random-effects (Hartung–Knapp) models. Squares represent study-specific HRs (sized by weight), with 95% CIs shown as horizontal lines; the diamond indicates the pooled estimate. HR < 1 indicates protection. Heterogeneity was assessed using I^2^ and τ^2^

Four RTS,S trials (n = 3470) provided hazard ratios (HR). Included trials show HR < 1, favoring RTS, S: Sacarlal et al. (2009): HR 0.69 (0.60–0.81), Aide et al. (2010): HR 0.74 (0.50–1.10), Olotu et al. (2011): HR 0.61 (0.47–0.80) and Abdulla et al. (2013): HR 0.65 (0.39–1.09). Under a random-effects model the pooled HR was 0.68 (95% CI 0.61–0.76), p = 0.0015, indicating 32% protection (95% CI 24–39%). There was no heterogeneity (I^2^ = 0%, τ^2^ = 0, p = 0.82), indicating that results are highly consistent across trials (Fig. 2). Leave-one-out analysis yielded point estimates ranging from 0.65 to 0.70, with no single study exerting undue influence.

### Efficacy against multiple episodes (Incidence-Rate Ratio, IRR)

**Fig. 3:**
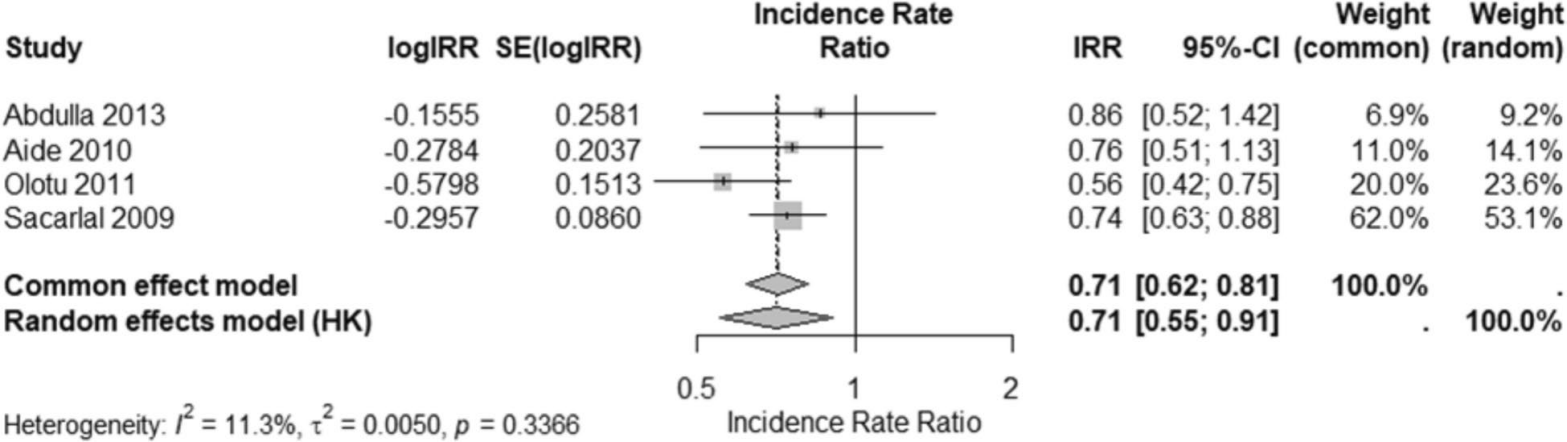
Forest plot of incidence-rate ratios (IRR) for multiple episodes of malaria comparing vaccinated and control groups across included randomized controlled trials. Pooled estimates were calculated using fixed-effect and random-effects (Hartung–Knapp) models. Squares represent study-specific IRRs (sized by weight), with 95% CIs shown as horizontal lines; the diamond indicates the pooled estimate. IRR < 1 indicates protection. Heterogeneity was assessed using I^2^ and τ^2^

Four RTS,S trials reported IRR (n = 3470). All individual estimates favour the vaccine: Sacarlal et al., (2009): IRR 0.74 (0.63–0.88), Abdulla et al., (2013): IRR 0.86 (0.52–1.42), and. The random-effects estimate was 0.71 (95% CI 0.55–0.91) indicating 29% protection against multiple malaria episodes (Fig. 3). Heterogeneity was low (I^2^ = 11.3%, τ^2^ = 0.0050, p = 0.34), showing good consistency across trials. Leave-one-out analysis gave pooled IRRs ranging from 0.68 to 0.75, with Olotu 2011 exerting the largest single influence.

### Efficacy against multiple episodes (Risk Ratios, RR)

**Fig. 4:**
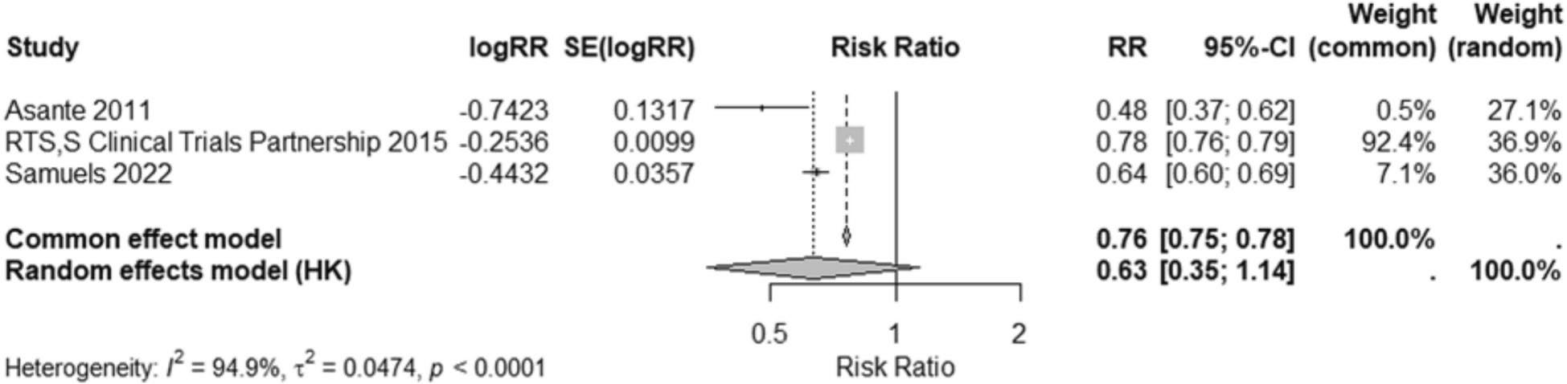
Forest plot of risk ratios (RR) for multiple episodes of malaria comparing vaccinated and control groups across included randomized controlled trials. Pooled estimates were calculated using fixed-effect and random-effects (Hartung–Knapp) models. Squares represent study-specific IRRs (sized by weight), with 95% CIs shown as horizontal lines; the diamond indicates the pooled estimate. IRR < 1 indicates protection. Heterogeneity was assessed using I^2^ and τ^2^

Three phase-III/IV RTS,S/AS01 trials Asante et al. (2011); RTS,S Clinical Trials Partnership (2015) and Samuels et al. (2022) contributed data on 17,579 participants. Individual trial risk ratios all favoured vaccination: 0.48 (95% CI 0.37–0.62), 0.78 (0.76–0.79) and 0.64 (0.60–0.69), respectively. Under a common-effect model the pooled RR was 0.76 (0.75–0.78), indicating 24% protection, but between-study heterogeneity was high (I^2^ = 94.9%, τ^2^ = 0.047, p < 0.0001). A random-effects model yielded a pooled RR of 0.63 (0.35–1.14). Although the point estimate suggests a 37% reduction in risk, the 95% confidence interval crosses 1.0, meaning it includes the possibility of no effect. Therefore, we cannot rule out that there is actually no difference between the groups. Leave-one-out sensitivity analysis produced pooled estimates ranging from 0.57 to 0.71, with no single study exerting disproportionate influence, yet heterogeneity remained high (I^2^ 79–97%) in every iteration (Fig. 4).

### Efficacy against severe malaria

**Fig. 5:**
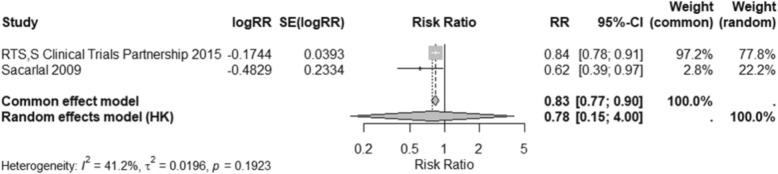
Forest plot of risk ratios (RR) for severe malaria comparing vaccinated and control groups across included randomized controlled trials. Pooled estimates were calculated using fixed-effect and random-effects (Hartung–Knapp) models. Squares represent study-specific IRRs (sized by weight), with 95% CIs shown as horizontal lines; the diamond indicates the pooled estimate. IRR < 1 indicates protection. Heterogeneity was assessed using I^2^ and τ^2^

Two trials (RTS,S Clinical Trials Partnership 2015 and Sacarlal 2009) provided data on severe malaria. Individual risk ratios were 0.84 (95% CI 0.78–0.91) and 0.62 (0.39–0.97), both favouring vaccination respectively. The common-effect pooled RR was 0.83 (0.77–0.90), corresponding to 17% protection, with moderate but non-significant heterogeneity (I^2^ = 41%, τ^2^ = 0.020, p = 0.19). Under a random-effects model the pooled RR remained 0.78, yet the 95% CI became wide (0.15–4.00). Leave-one-out sensitivity yielded estimates of 0.62 or 0.84 when each study was sequentially removed, confirming that the summary effect is chiefly determined by the larger 2015 trial and that the precision of the pooled estimate for severe malaria is limited (Fig. 5).

### Efficacy of R21 vaccine

A single phase-3 trial (n = 5 139) found an HR of 0.32 (95% CI 0.26–0.39) against the first episode (68% efficacy). The same trial reported IRR 0.33 (95% CI 0.27–0.41) against multiple episodes and HR 0.33 (95% CI 0.10–1.04) against severe malaria.

### Safety of the malaria vaccines

Across all included trials, malaria vaccine candidates demonstrated favorable safety profiles with no direct vaccine-attributable deaths or withdrawals due to vaccine adverse events. The R21/Matrix-M vaccine demonstrated a SAE rate of 3.8% (95% CI 3.4–4.3). A random-effects model of the two included trials enrolling 3,703 participants, gave a pooled SAE incidence of 3% (95% CI 1–6%; I^2^ = 80.6%).

**Fig. 6:**
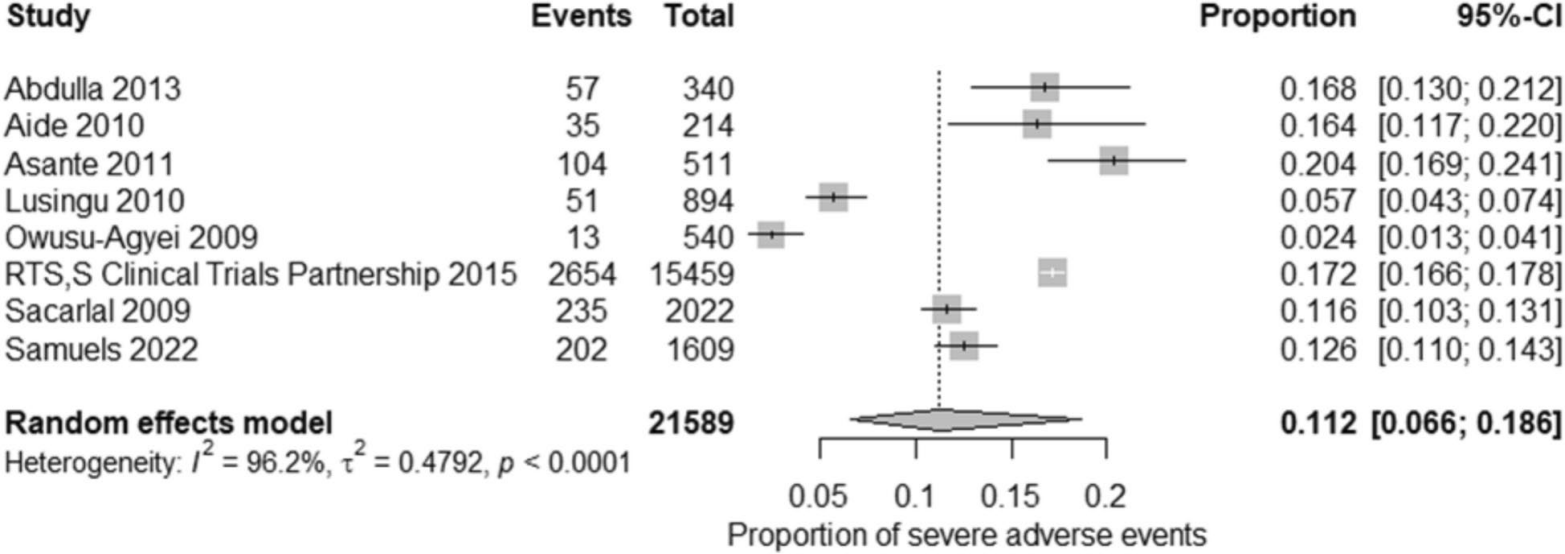
Forest plot – Proportion of participants with ≥ 1 SAE (RTS,S)

For RTS,S, the pooled serious adverse event rate ranged from 11.3% to 33.5% (weighted mean ~ 25%), compared to similar rates in control groups, indicating comparable safety profiles between malaria vaccine candidates and comparator vaccines (hepatitis B, rabies, or standard EPI vaccines). The RTS,S/AS01 vaccine, however, showed a meningitis safety signal in phase 3 testing. Febrile convulsions had an increased risk during the first 2–3 days post vaccination, but occurred at comparable rates when evaluated over a 30-day period in vaccine groups versus controls. Local and systemic reactogenicity was generally mild-to-moderate, with RTS,S/AS01 showing lower fever rates (11.3%) than rabies comparator (31.0%). A random-effects model, calculated from eight RTS,S trails enrolling a total of 21,589 participants, indicated a pooled SAE incidence of 11.2% (95% CI 6.6–18.6%), however, heterogeneity was high (I^2^ = 96.2%, τ^2^ = 0.48, p < 0.0001). Leave-one-out sensitivity analysis produced summary estimates between 10 and 14%, indicating that no single cohort exerted undue influence and that observed heterogeneity reflects real differences in surveillance intensity and case definitions across sites (Fig. 6).

### Quality and risk of bias assessment

**Fig. 7:**
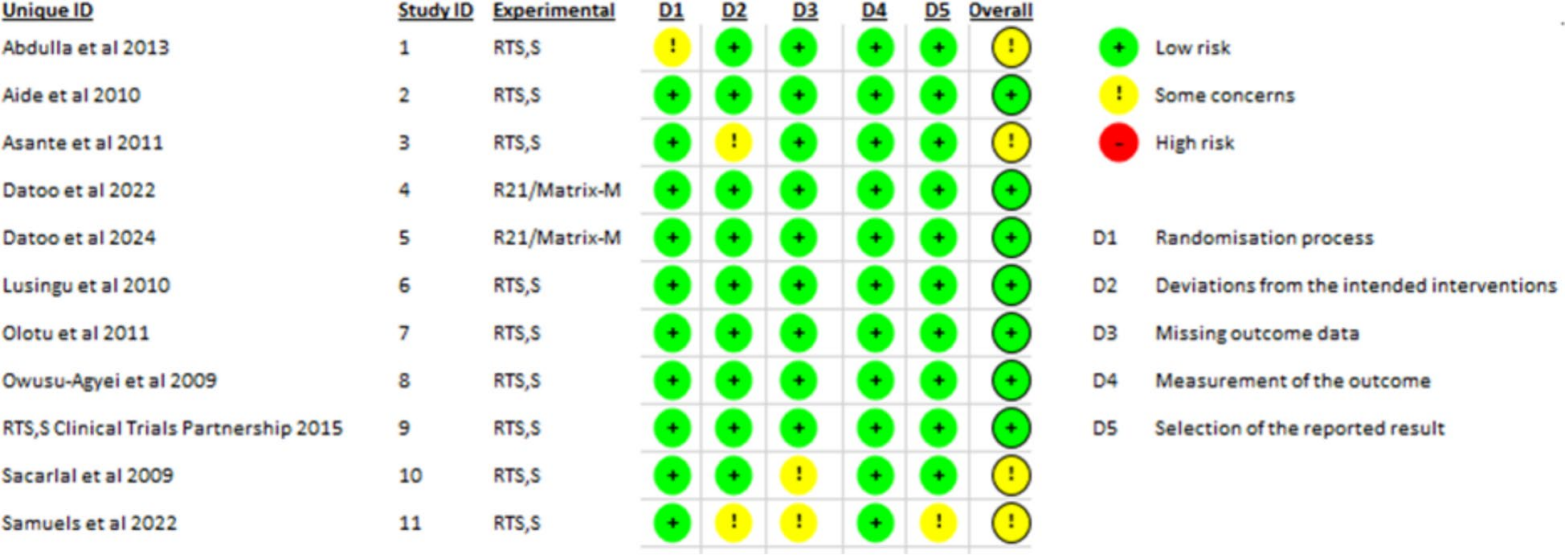
Traffic plot showing ROB-2 judgements for included studies

Risk-of-bias assessment with RoB-2 indicated that five of the nine RTS included reports had low risk of bias (Aide 2010, Lusingu 2010, Olotu 2011, Owusu-Agyei 2009, RTS,S Clinical Trials Partnership 2015). Both R21 trials were rated low risk (Datoo 2022 and Datoo 2024). Overall, the evidence base is at low risk of bias (Fig. 7).

## Discussion

The findings from this meta-analysis demonstrate that the RTS,S vaccine confers protection against malaria across multiple outcome measures, including time to first episode, recurrent malaria episodes, overall clinical malaria risk, and severe malaria. However, the magnitude and precision of protection have been shown to vary across the different trial phases and outcome definitions, with heterogeneity being a major interpretative consideration in large-scale studies.

In the early phase trials, protection estimates against single episode and recurrent malaria episodes were highly consistent. The pooled hazard ratio of 0.68 indicated a 32% reduction in the hazard of malaria with no heterogeneity being detected between the contributing studies (I^2^ = 0%). Similarly, the analysis of the recurrent malaria episodes yielded a pooled incidence rate ratio of 0.71, which corresponds to 29% protection against malaria with low to minimal heterogeneity (I^2^ = 11%). These findings suggest that under similar settings and conditions, the reduction in malaria risk produced by the RTS,S is reproducible. Furthermore, sensitivity analysis confirmed that these effects were not largely driven by any one single study, which reinforces the internal validity and reliability of early phase efficacy estimates.

However, in contrast later phase trials involving over 17,000 participants revealed major heterogeneity between studies (I^2^ = 95%). Despite the individual trials demonstrating statistically significant protection, the effect sizes demonstrated a wide range of risk reduction, varying from 22% to over 50%. Under a common effect model, pooled protection was estimated at 24%; however, when variability between studies was incorporated, the pooled point estimate remained protective but also imprecise. Furthermore, the leave-one-out analyses demonstrated that heterogeneity persisted irrespective of which study was removed, suggesting that there were methodological differences between the studies included rather than a single outlier influencing the overall result.

The evidence against protection of severe malaria, though promising, is very limited. Both included trials demonstrated a statistically significant reduction in malaria risk individually. However, because only two trials were included the estimation of variance between the studies resulted in very wide confidence intervals under a random effects model. Furthermore, sensitivity analyses confirmed that the larger phase III trial had a greater influence on the overall estimate.

Overall these findings support the following: the RTS,S vaccine reduces the malaria risk across the different outcomes measured; secondly, there is moderate yet reproducible protection against uncomplicated malaria, given controlled trial conditions; third, the magnitude of protection in large-scale implementation contexts varies substantially and finally, protection against severe malaria is promising however, unreliable due to the limited available data. With the current health conditions in Africa, even moderate reductions in malaria incidence could have a great impact in reducing morbidity and mortality amongst children under 5. Furthermore, of particular importance is the 29% reduction in recurrent episodes of malaria which is key in reducing malaria associated morbidity. This may relieve some of the burden of African healthcare systems especially in high transmission settings.

Despite all this, substantial heterogeneity in the phase III and IV trials limits adequate interpretation of the pooled averages suggesting that further exploration through subgroup analyses may be more informative. Additionally, given that only two studies despite having a large sample size were included, it constrains any inferences on the outcomes of severe malaria.

In contrast, the R21 vaccine demonstrated an efficacy of 68% protection against the first malaria episode and with similar strong reductions observed for recurrent malaria episodes and severe malaria. However this finding has limited statistical power because the confidence interval crosses 1.0. Whilst protection observed with the R21 is superior to the RTS,S trials caution must be taken as these findings were drawn only from a single study despite their large sample size and hence, the reproducibility of the R21 efficacy is yet to be established through further large scale implementation and diverse epidemiological contexts.

Our study also assessed the safety profiles of both RTS,S and R21/Matrix-M vaccines. In the RTS, S trials (n = 8), the pooled severe adverse events incidence (SAE) of the study population (n = 21,589) was 11.2% (95% CI 6.6–18.6). The SAE incidence should be interpreted with caution, given the high background morbidity rates in the areas where these trials were conducted. Many of the included trials were conducted in sub-Saharan Africa, where malaria transmission is relatively high. Therefore, the SAE rates may reflect increased background disease rather than vaccine-attributable harm.

The R21/Matrix-M vaccine was characterized by well-tolerated reactogenicity. In our study, the pooled SAE of the study population (n = 3703) was 3% (95% CI 1–6%; I^2^ = 80.6%). These findings are in accord with a multicentre phase 3 trial involving over 4,600 children in which SAEs were balanced between the R21/Matrix-M and the control arm (rabies vaccine) without treatment-related deaths [14]. Datoo et al. (2024) reported 142 serious adverse events (SAEs) and a higher frequency of local and systemic solicited adverse events, particularly injection site pain and fever. However, our study had a limited number of clinical trials (n = 2) to generate the R21 estimate. In addition, the absence of head-to-head trials between R21/Matrix-M and RTS, precludes a conclusion of superiority regarding safety. Continued surveillance is necessary to adequately determine the comparative safety profiles in wider populations.

## Conclusion

Malaria vaccines represent a leap in malaria prevention strategies in Africa. The substantial reduction in clinical episodes and severe disease, combined with favorable safety profiles, supports their integration into comprehensive malaria control strategies alongside insecticide-treated nets, chemoprevention, and improved case management. Real-world effectiveness monitoring will be essential to optimize their impact on child health in malaria-endemic sub-saharan Africa.

## Data Availability

Data is freely available upon request.
